# A study of the fine-structure constant dependence of radiative capture in Halo-EFT

**DOI:** 10.1140/epja/s10050-024-01408-1

**Published:** 2024-10-07

**Authors:** Ulf-G. Meißner, Bernard Ch. Metsch, Helen Meyer

**Affiliations:** 1 Helmholtz-Institut für Strahlen- und Kernphysik, Rheinische Friedrich-Wilhelms Universität Bonn, 53115 Bonn, Germany; 2 Bethe Center for Theoretical Physics, Rheinische Friedrich-Wilhelms Universität Bonn, 53115 Bonn, Germany; 3 Institute for Advanced Simulation (IAS-4), Forschungszentrum Jülich, 52425 Jülich, Germany

## Abstract

We study the fine structure constant dependence of the rates of some selected radiative capture reactions within the framework of so-called Halo Effective Field Theory in order to assess the adequacy of some assumptions made on the Coulomb penetrability. We find that this dependence deviates from that implied by a parameterization of the cross sections of this effect via a simple penetration factor. Some features of this fine-structure dependence are discussed, in particular its potential impact on the abundances of the light elements in primordial nucleosynthesis.

## Introduction

In Ref. [1] we made a re-assessment of the electromagnetic fine-structure constant dependence of the light element abundances in primordial nucleosynthesis or Big Bang nucleosynthesis (BBN). This required a description of the fine-structure constant dependence of the pertinent cross sections of the leading reactions in the BBN network. Only for the leading nuclear reaction, i.e. the radiative capture reaction $p + n \rightarrow d + \gamma$ a detailed and sufficiently accurate theoretical description within the framework of pionless Effective Field Theory (EFT) is available, see [3]. For the other reactions we accounted for the fact that the Q-value of a nuclear reaction, given as the difference of the total mass of the participating nuclei in the entrance and exit channel, depends on the value of the fine-structure constant, simply because the nuclear binding energies depend on the Coulomb interaction of the protons. Furthermore, we relied on a modeling of the Coulomb penetration factors in the form:

1 $$\begin{aligned} P(x) = \frac{x}{\text {e}^{x}-1} \end{aligned}$$

with

2 $$\begin{aligned} x = 2\pi \,\frac{Z_a\,Z_b\,\mu _{ab} c^2\,\alpha }{c\,p} = \sqrt{\frac{E_G(\alpha )}{E}} \end{aligned}$$

in terms of the so-called Gamow energy for a two-particle reaction channel *ij*

3 $$\begin{aligned} E_G(\alpha ) = 2\,\pi ^2\,Z_i^2\,Z_j^2\,\mu _{ij}  c^2\,\alpha ^2 \end{aligned}$$

and the center-of-mass (CMS) energy *E* or $E+Q$ for the entrance and the exit channel, respectively. Here, *p* is the corresponding CMS momentum, $Z_i$ the charge (in units of the elementary charge *e*) of nuclide *i*, $\mu _{ij}$ the reduced mass, *c* is the speed of light and $\alpha$ denotes the fine-structure constant. In addition, we accounted for a simple linear dependence on $\alpha$ in case of radiative capture reactions as well as a trivial $\alpha$ dependence reflecting the final momentum dependence if assuming dominance of dipole radiation, see [1]. We also noted in [1] that for some other radiative capture reactions an effective field theory description, viz. “Halo-EFT”, is available that potentially offers the possibility to study the $\alpha$ dependence of the cross sections analytically and thus assess the validity of the assumptions made in [1]. For a comprehensive overview on applications of Halo-EFT to nuclear structure and reactions we refer to the review [2]. The purpose of the present paper is to study the $\alpha$ dependence of the cross sections and the corresponding rates for the following radiative capture reactions: The neutron induced reaction

4 $$\begin{aligned} n +  ^{7}{\text {Li}} \rightarrow  ^{8}{\text {Li}} + \gamma , \end{aligned}$$

as treated in Refs. [4, 5], the proton induced reaction

5 $$\begin{aligned} p +  ^{7}{\text {Be}} \rightarrow  ^{8}{\text {B}} + \gamma , \end{aligned}$$

as treated in Ref. [6] and the two reactions that are most relevant to BBN:

6 $$\begin{aligned}  ^{3}{\text {H}} +  ^{4}{\text {He}} \rightarrow  ^{7}{\text {Li}} + \gamma , \end{aligned}$$

and

7 $$\begin{aligned}  ^{3}{\text {He}} +  ^{4}{\text {He}} \rightarrow  ^{7}{\text {Be}} + \gamma , \end{aligned}$$

as treated in Refs. [7, 8]. Although in the present contribution we shall rely on the implementation as elaborated in Refs. [4–8] we like to mention that the $n +  ^{7}{\text {Li}} \rightarrow  ^{8}{\text {Li}} + \gamma$ reaction was also treated within the Halo-EFT framework in Ref. [9]. Furthermore, earlier Halo-EFT work on the $p +  ^{7}{\text {Be}} \rightarrow  ^{8}{\text {B}} + \gamma$ reaction can be found in Refs. [10, 11]. For a discussion on the $^{3}{\text {He}} +  ^{4}{\text {He}} \rightarrow  ^{7}{\text {Be}} + \gamma$ reaction, we refer to [12].

The paper is organized as follows: In Sect. 2 we recapitulate the formulas for the radiative capture cross section in Halo-EFT. We then compare the results for the nominal $\alpha$ value with experimental data in Sect. 3. The results on the $\alpha$ dependence of the cross sections or astrophysical *S*-factors and the corresponding rates are discussed in Sect. 4. The impact on the changes of the light element abundances with a variation of the fine-structure constant is presented in Sect. 5. We summarize our findings in Sect. 6. Some technicalities not given in Refs. [6–8] are relegated to the Appendices.

## Basic formalism

Readers familiar with the framework of Halo-EFT as treated in Refs. [4–8] might directly proceed to Sect. 3. However, we find it useful to discuss the basic formalism as otherwise the discussion of the results becomes less transparent.

In Halo-EFT the nuclear system is assumed to consist of a “core”-system with mass $m_c$, charge number $Z_c$ and spin $s_c$ and a “valence”-system with mass $m_v$, charge number $Z_v$ and spin $s_v$. Furthermore, $M=m_c+m_v$ and $\mu =m_c\,m_v / M$ denote the total mass and the reduced mass of the system, respectively. With $L_i$ denoting the orbital angular momentum of the relative motion in the initial state, the total spin and total angular momentum in the initial state are given by $\vec {S}_i = \vec {s}_c+\vec {s_v}$ and ${\textbf{J}}_i = \vec {S}_i + \vec {L}_i$, respectively. Within the effective range expansion the initial state interaction is then specified by the parameters $a_{\zeta _i}$, $r_{\zeta _i}$, and $s_{\zeta _i}$, ($\zeta _i =  ^{S_i}{L_i}_{J_i}$) which for $L_i=0$ correspond to the *s*-wave scattering length, effective range and the first shape parameter, respectively.

The final state in the radiative capture reaction has mass $$M_f$$, charge number $$Z_f$$, excitation energy $$E_x$$ and total nuclear spin $$J_f$$. In terms of partial waves $$ ^{S_f}{L_f}_{J_f}$$ the final state is written as

8 $$\begin{aligned} \left| {M_f,E_x;J_f}\right\rangle= &   \sum _{s_f,L_f} a_{S_f,L_f}\,\left| {\left[ {\left[ {s_c}\!\times \!{s_v}\right] ^{S_f}}\!\times \!{L_f}\right] ^{J_f}}\right\rangle \nonumber \\= &   \sum _{\zeta } a_{\zeta _f}\,\left| {\zeta _f}\right\rangle \end{aligned}$$

with $S_f$ the total spin of the di-nuclear-cluster and $L_f$ the relative orbital angular momentum quantum number. The coefficients $a_{S_f,L_f}$ are the amplitudes for the decomposition of the final state in terms of di-nuclear states. Then

$$B_\zeta =M-(M_f+E_x)$$

is called the separation energy with respect to the clusters “*v*” and “*c*” and

$$\gamma _\zeta =\sqrt{2\,{\mu }\,B_\zeta }$$

the binding momentum of this state. We define

9 $$\begin{aligned} k_C = Z_c\,Z_v\,\alpha \,\mu \end{aligned}$$

as the inverse Bohr radius of a di-nuclear system in the case of charged particles.

### Electric dipole radiative capture

The formulas in this section are adopted from Ref. [8]. Assuming that the radiative capture proceeds through an electric dipole transition and that only a single state contributes, the cross section is given by the expression

10 $$\begin{aligned} \sigma _{E1}(p)= &   \frac{1}{16 \pi \,M^2}\,\frac{1}{(2s_c+1)(2s_v+1)} \nonumber \\  &   \times \sum _\zeta |a_\zeta |^2\,\frac{k^{(\zeta )}_\gamma }{p}\,\left| M_{E1}^{(\zeta )}\right| ^2, \end{aligned}$$

where *p* is the magnitude of the relative momentum in the CMS with $E=p^2/(2\mu )$ the non-relativistic expression for the energy of the relative motion and

11 $$\begin{aligned} k^{(\zeta )}_\gamma = \frac{p^2+\gamma _\zeta ^2}{2\,\mu } \end{aligned}$$

the non-relativistic approximation to the momentum of the photon in the final state.

The dimensionless amplitude squared reads

12 $$\begin{aligned} \left| M_{E1}^{(\zeta )}\right| ^2= &   64\pi \,\alpha \,(2J^\zeta _f+1)\,\frac{\left( Z_v m_c-Z_c m_v\right) ^2}{\mu \,\gamma _\zeta }\mathcal {N}(\eta ^\zeta _\gamma ,\rho ^\zeta _\gamma )\nonumber \\  &   \times \left[ \left| \mathcal {A}(p)\right| ^2+2\,\left| Y(p)\right| ^2\right] . \end{aligned}$$

Here, we defined $\eta ^\zeta _\gamma = k_C/\gamma _\zeta$ and $\rho ^\zeta _\gamma = \rho _1^\zeta /\gamma _\zeta$ with $\rho _1^\zeta = \hbar c/r_1^\zeta$ the effective momentum and $r_1^\zeta$ the effective range in the channel $\zeta$. The normalisation is given by

13 $$\begin{aligned}  &   {\mathcal {N}(\eta ,\rho )}=\frac{2\pi }{-\rho +4\,\eta \,h(\eta )+2\,\eta ^2\,(\eta ^2-1)\,h'(\eta )}, \end{aligned}$$

where

14 $$\begin{aligned} h(\eta ) = \psi (\eta ) + \frac{1}{2\,\eta }- \log (\eta ) \end{aligned}$$

and $\psi (\eta ) = \Gamma '(\eta )/\Gamma (\eta )$ is the digamma function. The normalisation is thus completely determined by the binding energy of the di-nuclear cluster (via $\eta$) and the effective momentum (via $\rho$) in the final state. In case of a neutral cluster $k_C = 0$ and thus $\eta = k_C/\gamma = 0$. Then

15 $$\begin{aligned} \left. \mathcal {N}(\eta ,\rho )\right| _{\eta =0}=-\frac{2\pi \,\gamma }{\rho +3\,\gamma }. \end{aligned}$$

With $\eta _p=k_C/p$ and $\kappa = \eta _p/\eta _Y=Y/p$ the capture from the initial *s*-wave is given by the amplitude

16 $$\begin{aligned} \frac{\left| \mathcal {A}(p)\right| }{C_0(\eta _p)}=\left| X(p)-\frac{2\pi }{\mu ^2}\, \frac{B(p) + \mu \,J_0(p)+\mu ^2\,k^{(\zeta )}_\gamma \,L^{(\zeta )}_{E_1}}{ \left[ C_0(\eta _p)\right] ^2\,p\left( \text {cotan}{\,}{(\delta _0)}-\text {i}\right) }\right| , \end{aligned}$$

where $L^{(\zeta )}_{E_1}$ is the low-energy constant of the two-body current contact term and

17 $$\begin{aligned} X(p)= &   1+ \frac{2}{3}\,\kappa \,\frac{\Gamma (2+\eta _\gamma )}{C_0(\eta _p)}\,\int _{0}^{\infty }\!\!\!\text {d}{\rho }\,\,W_{-\eta _\gamma ,\frac{3}{2}}\left( 2\,\kappa \,\rho \right) \,\nonumber \\  &   \times \left[ -\frac{F_0(\eta _p,\rho )}{\rho }+\partial _\rho F_0(\eta _p,\rho )\right] , \end{aligned}$$

is the *s*-wave contribution without initial state strong interactions in terms of Coulomb functions $F_\ell$ and Whittaker functions $W_{\eta ,\mu }$, which are the solutions to the pure Coulomb problem. In the case where $k_C=0$ (i.e. if a neutral particle is involved) this reduces to

18 $$\begin{aligned} \left. X(p)\right| _{\eta _\gamma =0}=1 - \frac{2}{3}\,\frac{p^2}{p^2+\gamma ^2}. \end{aligned}$$

The *s*-wave contribution from strong initial-state interactions is given by

19 $$\begin{aligned} \frac{B(p) + \mu \,J_0(p)}{\mu ^2\,p}= &   \frac{1}{3\pi }\,\frac{\text {i}-\kappa ^3}{1+\kappa ^2}+\eta _p\,\frac{C(p)}{\mu ^2} + \frac{\Delta B(p)}{\mu ^2\,p}\nonumber \\  &   -\frac{\eta _p}{2\pi }\left[ 2\,h(\text {i}\,\eta _p) + 2\,\gamma _E - \frac{5}{3}+\log {(4\pi )}\right] .\nonumber \\ \end{aligned}$$

Here, the function *C*(*p*) is given by a double integral, treated in App. A and the finite contribution ${\Delta B}(p)$ is evaluated as follows: The integrand

20 $$\begin{aligned} \mathcal {B}(\kappa ,\eta _\gamma ;\rho )= &   -\frac{\kappa }{3\pi }\Gamma (2+\eta _\gamma )\,\Gamma (1+\text {i}\,\kappa \,\eta _\gamma )\,W_{-\eta _\gamma ,\frac{3}{2}}\left( 2\,\kappa \,\rho \right) \,\nonumber \\  &   \times \,\left[ -\frac{1}{\rho }\,W_{-\text {\tiny i}\kappa \eta _\gamma ,\frac{1}{2}}(-2\,\text {i}\,\rho )+\partial _\rho W_{-\text {\tiny i}\kappa \eta _\gamma ,\frac{1}{2}}(-2\,\text {i}\,\rho )\right] \nonumber \\ \end{aligned}$$

in

21 $$\begin{aligned} \frac{B(p)}{\mu ^2\,p}=\int _{0}^{\infty }\!\!\!\text {d}{\rho }\,\,\mathcal {B}(\kappa ,\eta _\gamma ;\rho ), \end{aligned}$$

is quadratically divergent for $\rho \rightarrow 0$. Noting that the integrand depends on $\alpha$ via $\eta _\gamma = k_C/\gamma$ and $k_C \propto \alpha$, the integrand can be regularized by subtracting the terms from zero and single photon contributions, i.e. the terms of $\mathcal {O}(\alpha ^0)$ and $\mathcal {O}(\alpha ^1)$. Then, with

$$\begin{aligned} \alpha \,\frac{\partial  {\mathcal {B}(\alpha )}}{\partial {\alpha } }=\eta _\gamma \,\frac{\partial  {\mathcal {B}(\kappa ,\eta _\gamma ;\rho )}}{\partial {\eta _\gamma } }, \end{aligned}$$

the finite contribution^1^ is given by

22 $$\begin{aligned} \frac{{\Delta B}(p)}{\mu ^2\,p}= &   \int _{0}^{\infty }\!\!\!\text {d}{\rho }\,\,\Bigl [\mathcal {B}(\kappa ,\eta _\gamma ;\rho ) -\mathcal {B}(\kappa ,0;\rho )\nonumber \\    &   -\left( \partial _{\eta _\gamma }\mathcal {B}\right) (\kappa ,0;\rho )\cdot \eta _\gamma \Bigr ] \end{aligned}$$

which can be integrated numerically.^2^ For neutral particles, i.e. $k_C=0$, one obtains

23 $$\begin{aligned} \left. \frac{B(p)+\mu \,J_0(p)}{\mu ^2\,p}\right| _{k_C=0}=\frac{1}{3\pi }\,\frac{\text {i}-\kappa ^3}{1+\kappa ^2}-\frac{\text {i}}{2\pi }. \end{aligned}$$

Finally, the contribution from the initial *d*-wave states to the capture process is given by the amplitude

24 $$\begin{aligned} Y(p)= &   \frac{2}{3}\,\kappa \,\Gamma (2+\eta _\gamma )\,\int _{0}^{\infty }\!\!\!\text {d}{\rho }\,\,W_{-\eta _\gamma ,\frac{3}{2}}\left( 2\,\kappa \,\rho \right) \,\nonumber \\  &   \times \left[ \frac{2\,F_2(\eta _p,\rho )}{\rho }+\partial _\rho F_2(\eta _p,\rho )\right] \end{aligned}$$

which for $k_C=0$ reduces to

25 $$\begin{aligned} \left. Y(p)\right| _{k_C=0}=\frac{2}{3}\,\frac{p^2}{p^2+\gamma ^2}. \end{aligned}$$

### Magnetic dipole radiative capture

In case of single nucleon radiative capture there are additional relevant contributions from magnetic dipole transitions to the final states.

#### Neutron induced magnetic dipole contribution

In case of the $n +  ^{7}{\text {Li}} \rightarrow  ^{8}{\text {Li}} + \gamma$ reaction we recapitulate the formulas from Ref. [5]. Earlier work on this reaction, including the *M*1 contribution, can be found in Ref. [4].

The cross section for the *M*1 contribution to the radiative capture in the $^{7}{\text {Li}} + n \rightarrow  ^{8}{\text {Li}} + \gamma$ reaction through the $3^+$ resonance according to Ref. [4] is given by

26 $$\begin{aligned} \sigma _{M1}(p)= &   \frac{1}{14}\,\frac{7}{3}\,\frac{\alpha \,\mu }{m_p^2}\,\left| h^2\,\mathcal {Z}^{(\zeta )}\right| \,\left( \frac{k}{p}\right) ^3\,\nonumber \\  &   \times \left| \frac{p^2}{-\frac{1}{a_1^{(3)}+\frac{1}{2}\,r_1^{(3)}\,p^2}-\text {i}\,p^3}\right| ^2\nonumber \\    &   \times \Biggl \lbrace \left| \frac{2}{3}\,\frac{\gamma ^3-\text {i}\,p^3}{\gamma ^2+p^2}\,K^{(2)} + \beta ^{(2)}\right| ^2\nonumber \\  &   +\left| \frac{2}{3}\,\frac{\gamma ^3-\text {i}\,p^3}{\gamma ^2+p^2}\,K^{(1)} + \beta ^{(1)}\right| ^2\Biggr \rbrace , \end{aligned}$$

with $m_p$ the proton mass. The asymptotic normalization of the final $^{8}{\text {Li}}$-states ($\zeta =2^+$ for the ground state or $\zeta =1^+$ for the first excited state) with binding momentum $\gamma ^{(\zeta )}$ is given by

$$\begin{aligned} h^2\,\mathcal {Z}^{(\zeta )} = -\frac{2\pi }{3\,\gamma ^{(\zeta )}+r_1^{(\zeta )}}, \end{aligned}$$

the gyro-magnetic factors in the $^{5}{P}_{3} \rightarrow  ^{3}{P}_{2}$ and the $^{5}{P}_{3} \rightarrow  ^{5}{P}_{2}$ *M*1-transitions are given by

27 $$\begin{aligned} K^{(1)}= &   \sqrt{\frac{3}{2}}\left( \frac{3}{2}\,g_c - \frac{3}{2}\,g_v\right) ,\nonumber \\ K^{(2)}= &   \sqrt{\frac{3}{2}}\left( \frac{3}{2}\,g_c + \frac{1}{2}\,g_v+2\,\mu \,m_n\,\frac{Z_c}{m_c^2}\right) , \end{aligned}$$

in terms of the gyro-magnetic ratios $$g_c$$ and $$g_v$$ describing the magnetic moments of the core and the valence system, respectively, and $$\beta ^{(i)}, i=1,2$$ are constants reflecting the two-body current terms. Finally, $$a_1^{(3)}$$ and $$r_1^{(3)}$$ are the scattering volume and the effective momentum in the $$ ^{5}{P}_{3}$$ scattering channel.

#### Proton induced magnetic dipole contribution

This section summarizes the results quoted in Ref. [6] for the *M*1 contribution in the reaction

$$\begin{aligned}  ^{7}{\text {Be}} + p \rightarrow  ^{8}{\text {B}}(2^+) + \gamma , \end{aligned}$$

through the $1^+$ resonance. Considered is only the $^{5}{P}_{1} \rightarrow  ^{5}{P}_{2}$ transition assuming that the $1^+$ resonance is dominantly a proton ${p}_{\frac{1}{2}}$ coupled to the $^{7}{\text {Be}}(\frac{3}{2}^-)$ ground state with the amplitude

28 $$\begin{aligned} \left\langle {\left[ \frac{3}{2}^-\times \left[ \frac{1}{2}^+\times 1^-\right] ^\frac{1}{2}\right] ^1}\right| \left. { \left[ \left[ \frac{3}{2}^-\times \frac{1}{2}^+\right] ^2\times 1^-\right] ^1}\right\rangle =\sqrt{\frac{5}{6}}. \end{aligned}$$

The cross section for a magnetic dipole radiative reaction through the $1^+$ resonance is then given by [6]

29 $$\begin{aligned} \sigma _{M1}(p)=\frac{1}{16\pi \,M^2}\,\frac{1}{6}\sum _\zeta \frac{(k_\gamma ^{(\zeta )})^3}{p^3}\,\left| M^{(\zeta )}_{M1}\right| ^2. \end{aligned}$$

The squared matrix element reads

30 $$\begin{aligned} \left| M^{(\zeta )}_{M1}\right| ^2= &   (2J^{(\zeta )}_f+1)\,\mu ^3\,\frac{8\pi \,M^2\,\alpha }{m_p^2}\,\frac{|\mathcal {A}_1(p)|^2}{|C_1(\eta _p)|^2}\,\nonumber \\  &   \times \frac{2\pi }{\mu }\,\mathcal {Z}^{( ^{5}{P}_{2})}\,\frac{\mu ^2}{432\,\pi ^4}\,\left| \overline{L}_{22}(p)\right| ^2, \end{aligned}$$

where

31 $$\begin{aligned} \overline{L}_{22}(p)= &   \frac{2\pi }{\mu }\left\{ \frac{9\pi }{\sqrt{40}}\Biggl [3\,g_c + g_v+ 4\,\mu \,m_p\left( \frac{Z_c}{m_c^2} + \frac{Z_v}{m_v^2}\right) \right] \nonumber \\  &   \times \frac{\overline{D}(p,\gamma _\zeta )}{\mu ^2}-\beta _{22}\Biggr \rbrace \end{aligned}$$

with $\mathbf {\mu }_c = g_c\,\vec {j}_c$ and $\mu _v = g_v\,\vec {j}_v$ the (spin) magnetic moments of the constituents *c*, *v* of the di-nuclear system and

$$\begin{aligned} \mathbf {\mu }_L = \mu \,m_p\left( \frac{Z_c}{m_c^2} + \frac{Z_v}{m_v^2}\right) \,\textbf{L} \end{aligned}$$

the magnetic moment due to the current associated with the relative orbital motion.^3^

Furthermore,

32 $$\begin{aligned} \frac{\overline{D}(p)}{\mu ^2\,\gamma }= \frac{1}{3\pi }\,\frac{\text {i}\,\kappa ^3-1}{1+\kappa ^2} +\eta _\gamma \,\frac{D'(\eta _p,\kappa )}{\mu ^2} +\frac{\Delta D(k_C;p,\gamma )}{\mu ^2\,\gamma }, \end{aligned}$$

where we defined $\rho = p\,r, \eta _\gamma = {k_C}/{\gamma }, \kappa ={\gamma }/{p}, \eta _p = \kappa \,\eta _\gamma = {k_C}/{p}$. The integrand

33 $$\begin{aligned} \mathcal {D}(\kappa ,\eta _\gamma ;\rho )= &   -\text {i}\,\frac{\kappa }{3\pi }\, \Gamma (2+\text {i}\,\kappa \,\eta _\gamma )\,\Gamma (2+\eta _\gamma )\nonumber \\  &   \times {W}_{-\eta _\gamma ,\frac{3}{2}}(2\,\kappa \,\rho )\,W_{-\text {i}\,\eta _p,\frac{3}{2}}(-2\,\text {i}\,\rho ) \end{aligned}$$

in

34 $$\begin{aligned} D(k_C;p,\gamma ) = \mu ^2\,p\,\int _{0}^{\infty }\!\!\!\text {d}{\rho }\,\mathcal {D}(\kappa ,n_\gamma ;\rho ) \end{aligned}$$

is again divergent for $\rho \rightarrow 0$. By subtracting the zero and single photon contributions one then defines the finite term

35 $$\begin{aligned} \Delta D(k_C;p,\gamma )= &   \mu ^2\,p\,\int _{0}^{\infty }\!\!\!\text {d}{\rho }\Bigl [\mathcal {D}(\kappa ,\eta _\gamma ;\rho ) -\mathcal {D}(\kappa ,0;\rho )\nonumber \\    &   -\left( \partial _{\eta _\gamma }\mathcal {D}\right) (\kappa ,0;\rho )\cdot \eta _\gamma \Bigr ]. \end{aligned}$$

The evaluation of the second term $D'(\eta _p,\kappa )$ on the r.h.s. of Eq. (32) is given in Appendix B.

The initial-state interaction in the $$ ^{5}{P}_{1}$$ channel is given by the amplitude

36 $$\begin{aligned}  &   \gamma ^2\,\mathcal {A}_1(p)= \frac{2\pi \,\gamma }{\mu }\nonumber \\  &   \quad \times \frac{9\,(C_1(\eta _p))^2\,\text {e}^{\text {\tiny i}\,2\,\sigma _1}}{-\frac{1}{a_1^{( ^{5}{P}_{1})}\,p^2\,\gamma } + \frac{1}{2}\,\frac{r^{( ^{5}{P}_{1})}}{\gamma }\, -2\,\frac{p}{\gamma }\,\eta _p\,(\eta _p^2+1)\,H(\eta _p)},\nonumber \\ \end{aligned}$$

where $a^{( ^{5}{P}_{1})}$ is the scattering volume and $r^{( ^{5}{P}_{1})}$ the effective momentum to reproduce the $1^+$ resonance with position $E_R=p_R^2/(2\mu ) = 0.630(3) \text { MeV}$ and width $\Gamma _R = 0.0357(6) \text { MeV}$. The normalization of the final state is determined by

37 $$\begin{aligned}  &   \gamma \,\frac{2\pi }{\mu }\mathcal {Z}^{( ^{5}{P}_{2})}\nonumber \\  &   \quad =\frac{2\pi }{-\rho _\gamma + 2\,\eta _\gamma ^2(\eta _\gamma ^2-1)\,h'(\eta _\gamma )+4\,\eta _\gamma \,h(\eta _\gamma )} \end{aligned}$$

with $$\rho _\gamma = {r_1^{( ^{5}{P}_{2})}}/{\gamma }$$.

## Cross sections, astrophysical S-factors and reaction rates.

The cross sections $\sigma$ or the corresponding astrophysical *S*-factors given as

38 $$\begin{aligned} S(E) = E\,\sigma (E)\,\text {e}^{\sqrt{E_G/E}} \end{aligned}$$

with $E_G$ the Gamow energy in the entrance channel, were calculated according to the formulas given in the previous section, Sect. 2. The nuclear structure parameters are given in Table 1. The values of the electromagnetic contribution to the nuclear binding energies $V_C$ were obtained from an ab initio calculation within the framework of Nuclear Lattice EFT, see Table 1 in Ref. [1]. Furthermore, the reaction parameters for the nucleon induced reactions are given in Table 2 and for the radiative capture to $^{7}{\text {Li}}$ and $^{7}{\text {Be}}$ are given in Table 3.

**Table 1:** Nuclear structure data: Nuclear mass in MeV, spin/parity $J^\pi$, excitation energy $E_x$ in MeV, binding momentum $\gamma$ with respect to the di-nuclear system in MeV, the gyro-magnetic ratio *g* and the ground state nuclear Coulomb energy $V_C$ in MeV [1]

Nucleus	Mass	$J^\pi$	$E_x$	$\gamma$	*g*	$V_C$
*p*	938.2721	$\frac{1}{2}^+$	0	–	5.5857	0
*n*	939.5654	$\frac{1}{2}^+$	0	–	–3.8261	0
$^{3}{\text {H}}$	2808.9211	$\frac{1}{2}^+$	0	–	–	0
$^{3}{\text {He}}$	2808.3916	$\frac{1}{2}^+$	0	–	–	0.6884
$^{4}{\text {He}}$	3727.3794	$0^+$	0	–	–	0.7588
$^{7}{\text {Li}}$	6533.8330	$\frac{3}{2}^-$	0	88.9099	2.1710	1.5994
		$\frac{1}{2}^-$	0.4776	79.8429	–	–
$^{7}{\text {Be}}$	6534.1841	$\frac{3}{2}^-$	0	71.2970	$-$0.9329	2.7117
		$\frac{1}{2}^-$	0.4291	60.8999	–	–
$^{8}{\text {Li}}$	7471.3658	$2^+$	0	57.7872	–	1.6491
		$1^+$	0.9808	41.5694	–	–
$^{8}{\text {B}}$	7472.3201	$2^+$	0	14.9465	–	4.2119

**Table 2:** Reaction parameters for the nucleon induced radiative capture reactions: *s*-wave scattering length $a_0$ in fm, *p*-wave scattering volume $a_1$ in $\hbox {fm}^3$, *p*-wave effective momentum $r_1$ in MeV, two-body current parameter $\beta ^{(1)}=\beta ^{(2)} = \beta$ for the neutron induced reaction, $\beta =\beta _{22}$ for the proton induced reaction, both in MeV. $r_1( ^{5}{P}_{2})^*$ is the value used in calculating the *M*1 contribution. For the $^{7}{\text {Li}}(n,\gamma ) ^{8}{\text {Li}}$ reaction the parameter sets “A” and “ANC” correspond to those called “EFT A” and “EFT ANC” in Ref. [5], respectively. For the $^{7}{\text {Be}}(p,\gamma ) ^{8}{\text {B}}$ reaction the parameter sets “NNLO” and “ANC” correspond to those called “$\text {EFT}_{\text {\tiny gs}}$ I NNLO” and those related to a determination from A(symptotic) N(ormalization) C(oefficients), respectively, see Ref. [6]

Parameter	$^{7}{\text {Li}}(n,\gamma ) ^{8}{\text {Li}}$		$^{7}{\text {Be}}(p,\gamma ) ^{8}{\text {B}}$	
	A	ANC	NNLO	ANC
$a_0( ^{3}{S}_{1})$	0.87	0.87	17.34	17.34
$a_0( ^{5}{S}_{2})$	$-$ 3.63	$-$ 3.63	$-$3.18	$-$ 3.18
$r_1( ^{3}{P}_{2})$	$-$ 290.0707	$-$ 605.6995	$-$173.0	$-$ 176.8
$r_1( ^{5}{P}_{2})$	$-$ 290.0707	$-$ 270.0028	$-$32.92	$-$ 40.31
$r_1( ^{5}{P}_{2})^*$	$-$ 290.1	$-$ 290.1	$-$30.00	$-$ 30.00
$r_1( ^{3}{P}_{1})$	$-$ 473.5848	$-$ 638.1095		
$a_1( ^{5}{P}_{1})$			$-$108.13	$-$108.13
$r_1( ^{5}{P}_{1})$	$-$ 473.5848	$-$ 498.0909	$-$111.23	$-$ 111.23
$a_1( ^{5}{P}_{3})$	$-$ 77.0136	$-$ 547.1		
$r_1( ^{5}{P}_{3})$	$-$ 77.0136	$-$ 547.1		
$\beta$	170.0	170.0	375.0	375.0

**Table 3:** Reaction parameters for the $^{3}{\text {H}} +  ^{4}{\text {He}} \rightarrow  ^{7}{\text {Li}} + \gamma$ and $^{3}{\text {He}} +  ^{4}{\text {He}} \rightarrow  ^{7}{\text {Be}} + \gamma$ reactions: The *s*-wave scattering length $a_0$ in fm, the *s*-wave effective range $r_0$ in fm, the *s*-wave shape parameter $s_0$ in $\hbox {fm}^3$, the *p*-wave effective momentum $r_1$ in MeV, the *p*-wave shape parameter $s_1$ in fm and the LEC. The parameter sets labeled ‘fit”, “A” and “fit”,“AII” correspond to those labeled “$\chi ^2$”,“Model A” and “$\chi ^2$” and “Model AII” in Ref. [8] for these two reactions, respectively

Parameter	$^{4}{\text {He}}( ^{3}{\text {H}},\gamma ) ^{7}{\text {Li}}$		$^{4}{\text {He}}( ^{3}{\text {He}},\gamma ) ^{7}{\text {Be}}$	
	fit	A	fit	AII
$a_0( ^{2}{S}_{\frac{1}{2}})$	17.0	13.0	22.0	40.0
$r_0( ^{2}{S}_{\frac{1}{2}})$	0.6	$-$ 0.1	1.2	1.09
$s_0( ^{2}{S}_{\frac{1}{2}})$	2.0	11.0	$-$ 0.9	$-$ 2.2
$r_1( ^{2}{P}_{\frac{1}{2}})$	$-$ 129.0	$-$ 230.0	$-$ 41.9	$-$ 45.0
$r_1( ^{2}{P}_{\frac{3}{2}})$	$-$ 149.0	$-$ 190.0	$-$ 55.4	$-$ 59.0
$s_1( ^{2}{P}_{\frac{1}{2}})$	–	–	1.74	1.84
$s_1( ^{2}{P}_{\frac{3}{2}})$	–	–	1.59	1.69
LEC$( ^{2}{P}_{\frac{1}{2}})$	1.5	4.0	0.83	1.07
LEC$( ^{2}{P}_{\frac{3}{2}})$	1.44	2.2	0.78	1.02

The reaction rate in thermal equilibrium at temperature *T* then follows from the cross section via

39 $$\begin{aligned} \gamma (T) = N_A\sqrt{\frac{8}{\pi \,\mu \,(k_B T)^3}}\int _{0}^{\infty }\!\!\!\text {d}{E}\,\,\,\sigma (E)\,E\,\text {e}^{-\frac{E}{k_B T}}\,, \end{aligned}$$

where $N_A$ is the Avogadro number and $k_B$ the Boltzmann constant. Essentially, this expression for the rate has the form of a Laplace-transform of the cross section multiplied by the CM-energy.

In the next subsections we present the resulting cross sections or astrophysical *S*-factors as well as the corresponding rates according to Eq. (39) for the four reactions studied here, all calculated at the present value of the fine-structure constant that we shall call the nominal value of $\alpha$ given by

40 $$\begin{aligned} \alpha _0= &   7.2973525693(11) 10^{-3}\nonumber \\  = &   1/137.035999084(2) \end{aligned}$$

from Ref. [13]. For each of the reactions considered here we present the calculated rates for two parameter sets used in the Halo-EFT calculations in order to give an impression of the systematic uncertainty. In addition, we compare the resulting rates with those used in the original versions of some publicly available BBN codes: viz. NUC123 [14], AlterBBN [15, 16], PArthENoPE [17–19] and PRIMAT [20], if available. These were also considered in our study [1] mentioned in the introduction. The most recent code PRyMordial, see [21, 22], by default uses the PRIMAT-rates and thus in this context is not discussed separately.

### The $n +  ^{7}{\text {Li}} \rightarrow  ^{8}{\text {Li}} + \gamma$ reaction

In this case $$Z_v = 0, Z_c=3$$, $$s_v = 1/2, s_c={3}/{2}$$ and $$Z_f=4$$ with $$J_f = 2$$ for the ground state and $$J_f=1$$ for the excited state. The final $$2^+$$ state is supposed to be an equal mixture of the $$ ^{3}{P}_{2}$$ and $$ ^{5}{P}_{2}$$ states, i.e.

41 $$\begin{aligned} \left| {2^+}\right\rangle =\frac{1}{\sqrt{2}}\left| { ^{3}{P}_{2}}\right\rangle +\frac{1}{\sqrt{2}}\left| { ^{5}{P}_{2}}\right\rangle , \end{aligned}$$

while the excited $1^+$ state is supposed to be

42 $$\begin{aligned} \left| {1^+}\right\rangle = -\frac{1}{\sqrt{6}}\left| { ^{3}{P}_{1}}\right\rangle + \sqrt{\frac{5}{6}}\left| { ^{5}{P}_{1}}\right\rangle . \end{aligned}$$

The total radiative capture cross section in this case is given by the sum of the expression for the electric dipole contribution given in Eqs. (10, 12) with the special formulas for $k_C=0$ given in Eqs. (15, 18, 23, 25) and the magnetic dipole contribution of Eq. (26).

**Fig. 1:**
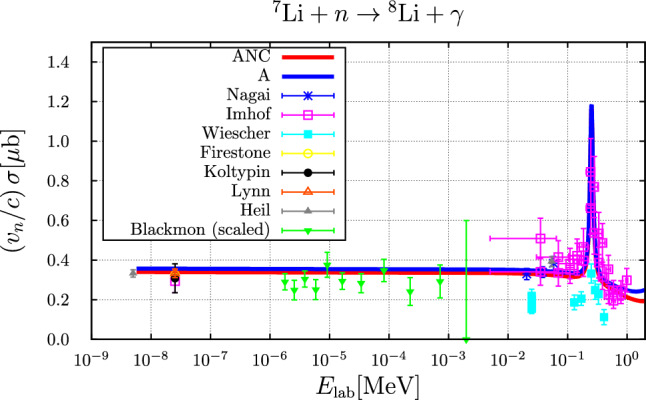
Cross section (scaled with the laboratory neutron velocity $v_n/c$) as a function of the laboratory neutron energy compared to experimental data: Nagai [23], Imhof [24], Wiescher [25], Firestone [26], Koltypin [27], Lynn [28], Heil [29], Blackmon [30]. The latter data were divided by the branching ratio 0.89 for the ground state in order to also account for the $1^+$ final state contribution such that these data now represent the total capture cross section

The resulting cross section (scaled with the laboratory neutron velocity) is compared to the experimental data in Fig. 1.

**Fig. 2:**
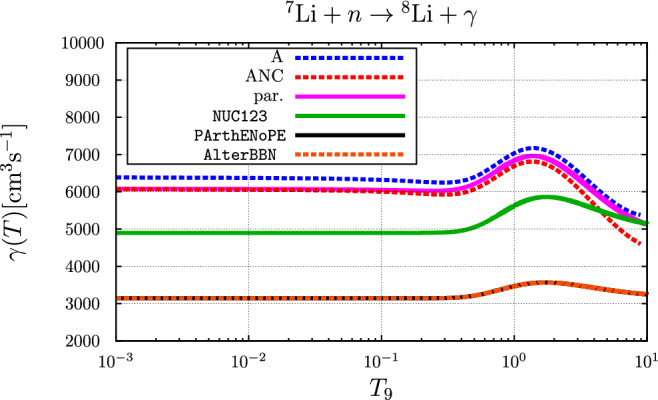
Comparison of the temperature-dependent rate for the $n+ ^{7}{\text {Li}} \rightarrow  ^{8}{\text {Li}} + \gamma$ reaction. Here, $T_9 = T / 10^9~\text {K}$. The blue and red curves represent the results from the parameter sets “A” and “ANC”, respectively, the purple curve is based on the parameterization of Eq. (43), the green curve is the rate used in NUC123 [14], the curves for the parameterizations in PArthENoPE [17–19] (solid black curve) and AlterBBN [15, 16] (dashed brown curve) are identical

The calculated rates for the parameter sets “A” and “ANC” (these correspond to the parameter sets called “EFT A” and “EFT ANC” in Ref. [5], respectively; “ANC” standing for: “parameters corresponding to empirical A(symptotic) N(ormalization) C(oefficient)”) are compared to the rates as parameterized in NUC123 [14], PArthENoPE [17–19] and AlterBBN [15, 16] as well as to the rate resulting from the following novel parameterization of the cross section, accounting for the $3^+$ resonance via a non-relativistic Breit–Wigner parameterization

43 $$\begin{aligned} \sqrt{E}\,\sigma (E)= &   0.0675\,\frac{1-0.045\,E+0.7\,E^2}{1+0.001\,E+0.7\,E^2}\nonumber \\  &   +\frac{0.018}{1+5000.0\,(E-0.2215)^2},\nonumber \\  &   \text {(in mb MeV} ^{\frac{1}{2}}, \text {with}\,\, E \,\, \text {in MeV)} \end{aligned}$$

in Fig. 2. Indeed this parameterization yields a rate very similar to those of the Halo-EFT calculation.

### The $p +  ^{7}{\text {Be}} \rightarrow  ^{8}{\text {B}} + \gamma$ reaction

In this case $$Z_v = 1, Z_c=4$$, $$s_v = 1/2, s_c={3}/{2}$$ and $$Z_f=5$$ with $$J_f = 2$$ for the ground state, which, as the corresponding ground state of the mirror nucleus, is supposed to be an equal mixture of the $$ ^{3}{P}_{2}$$ and $$ ^{5}{P}_{2}$$ states. The total radiative capture cross section is given by the sum of the expression for the electric dipole contribution in Eqs. (10,12) with $$k_C \ne 0$$ and the resonant magnetic dipole contribution as given in Eq. (29).

The resulting *S*-factor is compared to experimental data and to the parameterization

44 $$\begin{aligned} S(E)= &   0.018\,\frac{1+0.3\,E+0.125\,E^2}{1+0.017\,E^2}\nonumber \\  &   +\frac{0.090}{1+2500.0\,(E-0.63)^2},\nonumber \\  &   \text {(in MeV mb, with}\,\, E \,\, \text {in MeV)}. \end{aligned}$$

in Fig. 3.

**Fig. 3:**
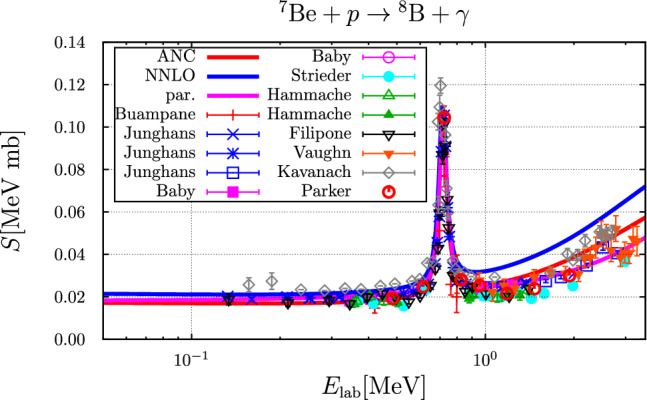
*S*-factor as a function of the laboratory proton energy compared to experimental data: Buompane [31], Junghans [32–35], Baby [36–38], Strieder [39], Hammache [40], Filipone [41, 42], Vaughn [43], Kavanach [44], Parker [45]. The blue and red curve correspond to the parameter sets “NNLO” and “ANC”, respectively. The purple curve represents the parameterization given in Eq. (44)

**Fig. 4:**
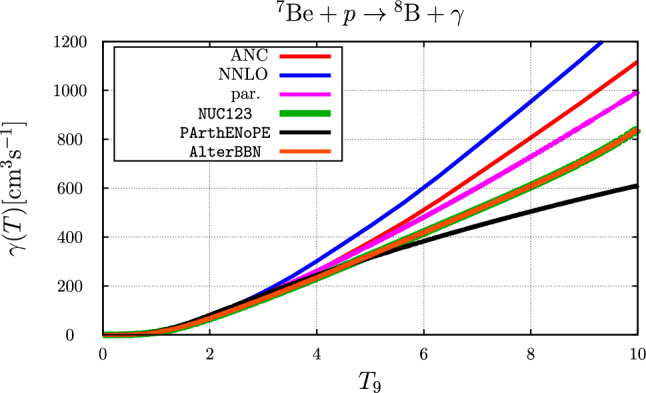
Comparison of the temperature ($T_9 = T / 10^9~\text {K}$) dependent rate for the $p+ ^{7}{\text {Be}} \rightarrow  ^{8}{\text {B}} + \gamma$ reaction. The blue and red curves represent the parameter sets “NNLO” and “ANC”, respectively, the purple curve is based on the parameterization of Eq. (44), the black curve is the rate used in PArthENoPE [17–19], the curves for the parameterizations in AlterBBN [15, 16] (brown curve) and NUC123 [14] (green curve) are identical

The calculated rate for the parameter sets “NNLO” and “ANC”, where these labels refer to the parameter sets labeled “$\text {EFT}_{\text {\tiny gs}}$ I NNLO” and those related to a determination from ANCs, respectively, see Ref. [6], are compared to the rates as parameterized in NUC123 [14], PArthENoPE [17–19] and AlterBBN [15, 16] as well as to the rate corresponding to the parameterization of the *S*-factor of Eq. (44) in Fig. 4.

### The $^{3}{\text {H}} +  ^{4}{\text {He}} \rightarrow  ^{7}{\text {Li}} + \gamma$ reaction

In this case $Z_v = 1, Z_c=2$, $s_v = 1/2, s_c=0$ and $Z_f=3$ with $J_f = {3}/{2}$ for the ground state and $J_f = {1}/{2}$ for the first excited state. The radiative capture cross section is determined by electric dipole contributions only, i.e. by the expression for the electric dipole contribution in Eqs. (10, 12) with $k_C \ne 0$. The parameter sets labeled “fit” and “A” correspond to the parameter sets labeled “$\chi ^2$” and “Model A” in Ref. [8], respectively.

**Fig. 5:**
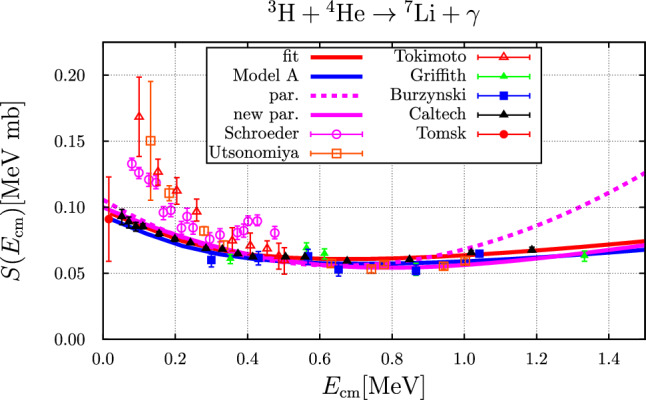
*S*-factor as a function of the centre-of-mass energy compared to experimental data: Schroeder [46], Utsonomiya [47–50], Tokimoto [51] (open symbols, corresponding to indirect (and thus reaction model dependent) determinations of the *S* factor from Coulomb break-up reactions; these are considered to be less reliable and shown here merely for completeness), Griffith [52], Burzynski [53], Caltech [54], Tomsk [55] (solid symbols, corresponding to direct measurements of the cross section), also see Ref. [55] for a comparison. The blue and red curve correspond to the parameter sets “A” and “fit”, respectively. The dotted purple curve represents the *S*-factor from the parameterization as used in Ref. [1]. The solid purple curve corresponds to the improved parameterization given in Eq. (45)

In Fig. 5 the S-factor for this reaction is compared to experimental data and to the parameterization used in Ref. [1]. Here we also compare to a new parameterization given as

45 $$\begin{aligned} S(E)= &   0.01\,\frac{1-1.15\,E+1.0\,E^2}{1+0.01\,E+0.5\,E^2}\nonumber \\  &   \text {(in MeV mb, with}\,\, E \,\, \text {in MeV)}. \end{aligned}$$

**Fig. 6:**
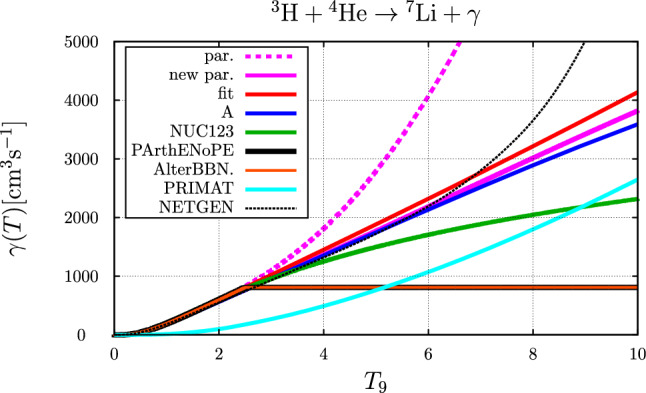
Comparison of temperature ($T_9 = T / 10^9~\text {K}$) dependent rates for the $^{3}{\text {H}}+ ^{4}{\text {He}} \rightarrow  ^{7}{\text {Li}} + \gamma$ reaction. The blue and red curve represents the parameter sets “A” and “fit”, respectively, the purple curve is the result corresponding to the parameterization of Eq. (45), while the dotted purple curve corresponds to the parameterization of Ref. [1]. Also shown are the parameterization of the rate as originally implemented in NUC123 [14] (green curve), PArthENoPE [17–19] (black curve), AlterBBN [15, 16] (brown curve) and PRIMAT [20] (cyan curve) as well as the parameterization provided by NETGEN, see [56]

This new parameterization of the *S*-factor is closer to the calculations within the framework of Halo-EFT studied here and improves the description of the data, in particular for energies $E_{\text {cm}}>1~\text {MeV}$, and indeed yields a rate that is much smaller at higher temperatures, see Fig. 6.

### The $^{3}{\text {He}} +  ^{4}{\text {He}} \rightarrow  ^{7}{\text {Be}} + \gamma$ reaction

In this case $Z_v = 2, Z_c=2$, $s_v = 1/2, s_c=0$ and $Z_f=4$ with $J_f = {3}/{2}$ for the ground state and $J_f = {1}/{2}$ for the first excited state. The radiative capture cross section is again determined by electric dipole contributions only, i.e. by the expression for the electric dipole contribution in Eqs. (10, 12) with $k_C \ne 0$. The parameter sets labeled “fit” and “AII” correspond to the parameter sets labeled “$\chi ^2$” and “Model AII” in Ref. [8], respectively.

**Fig. 7:**
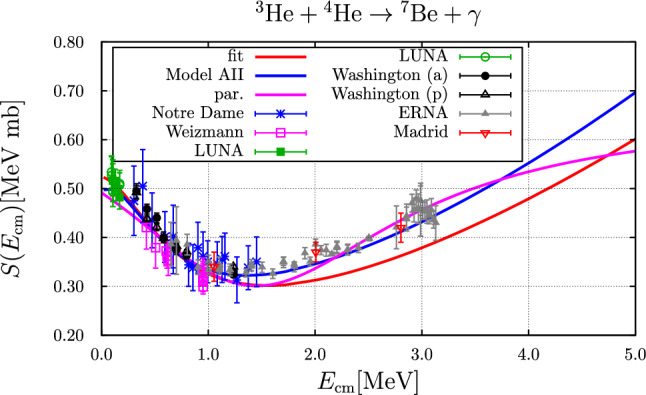
*S*-factor as a function of the centre-of-mass energy compared to experimental data: Notre Dame [57], Weizmann [58], LUNA [59, 60], Washington [61], ERNA [62], Madrid [63]. The blue and red curve correspond to the parameter sets “A II” and “fit”, respectively. The purple curve represents the parameterization as used in Ref. [1]

**Fig. 8:**
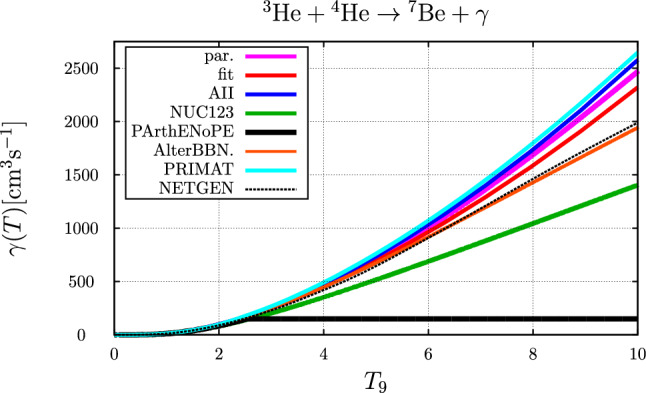
Comparison of temperature ($T_9 = T / 10^9~\text {K}$) dependent rates for the $^{3}{\text {He}}+ ^{4}{\text {He}} \rightarrow  ^{7}{\text {Be}} + \gamma$ reaction. The blue and red curve represent the parameter sets “AII” and “fit”, respectively, the purple curve corresponds to the parameterization of Ref. [1]. Also shown are the parameterizations of the rate as originally implemented in NUC123 [14] (green curve), PArthENoPE [17–19] (black curve), AlterBBN [15, 16] (brown curve) and PRIMAT [20] (cyan curve) as well as the parameterization provided by NETGEN, see [56]

The astrophysical *S*-factor is displayed in Fig. 7. The resulting nominal rates are given in Fig. 8.

## The fine-structure constant dependence of the rates

In order to study the fine-structure constant dependence we calculated the rates for

46 $$\begin{aligned} \alpha = \alpha _0\,(1+\delta ), \end{aligned}$$

and the fractional change in $\alpha$, i.e. $\delta$ was varied in the range $[-0.05,+0.05]$. We shall distinguish direct and indirect effects of the variation of the fine-structure constant:

Direct effect First of all, the fine-structure constant $\alpha$ enters the calculation of the radiative capture cross section as a linear factor due to the coupling of the electromagnetic field to the charges and currents, which in the amplitude is proportional to *e* and hence in the cross section leads to a proportionality $e^2 \propto \alpha$. Furthermore, $\alpha$ enters the cross section via the inverse Bohr-radius $k_C = Z_v\,Z_c\, \mu c^2\,\,\alpha$, that in turn determines the dimensionless quantities $\eta _\gamma = k_C/\gamma$, where $\gamma$ is the binding momentum, $\eta _\rho = k_C/\rho$, where $\rho$ the *p*-wave effective range, and $\eta _p = k_C/p$ (the Sommerfeld-parameter), that enter the expressions for the normalization $\mathcal {N}(\eta _\gamma ,\eta _\rho )$ (Eq. (13)), the amplitudes $\mathcal {A}(\eta _\gamma ;\eta _p)$ (Eq. (16)), via $X(\eta _\gamma ;\eta _p)$ (Eq. (17)), and $\mathcal {B}(\eta _\gamma ;\eta _p)$ (Eq. (20)), as well as $Y(\eta _\gamma ;\eta _p)$ (Eq. (24)). The Sommerfeld-parameter $\eta _p$ also enters the astrophysical *S*-factor.

Because the dependence of $k_C$ on $\alpha$ is linear, $k_C \propto \alpha$, we have

47 $$\begin{aligned} k_C(\alpha )=k_C(\alpha _0\,(1+\delta ))=k_C(\alpha _0)\,(1+\delta ). \end{aligned}$$

We shall call this the “direct effect”.

Indirect effect On top of this, the value of $\alpha$ influences the nuclear binding energies, i.e. the $\alpha$ dependence of the nuclear mass of the nuclide *i* is given by

48 $$\begin{aligned} m^i(\alpha )=m^i_N + V^i_C\,(1+\delta )=m^i + V^i_C\,\delta , \end{aligned}$$

where $V^i_C$ denotes the (repulsive) Coulomb-energy contribution to the nuclear mass. This in turn influences the *Q*-value of the reaction, i.e.

49 $$\begin{aligned} Q(\alpha )=m_v(\alpha ) + m_c(\alpha ) - M_f(\alpha ) = B_f(\alpha ) \end{aligned}$$

and thus the binding momentum

50 $$\begin{aligned} \gamma (\alpha )=\sqrt{2\,\mu (\alpha ) c^2\,B_f(\alpha )}. \end{aligned}$$

Concerning the kinematics of the reaction: for a given CMS kinetic energy *E*, the CMS relative momentum in the entrance channel *p* and the CMS final photon momentum $k_\gamma$ are given by

51 $$\begin{aligned} s(E)= &   (m_v + m_c + E)^2,\end{aligned}$$

52 $$\begin{aligned} p(E)= &   \sqrt{\frac{\left( s(E)-(m_c-m_v)^2\right) \left( s(E)-(m_c+m_v)^2\right) }{4\,s(E)}}\nonumber \\\approx &   \sqrt{2\,\mu \,E},\end{aligned}$$

53 $$\begin{aligned} k_\gamma (E)= &   \frac{s(E)-M_f^2}{\sqrt{4\,s(E)}}\approx Q + E \end{aligned}$$

and thus all depend on $\alpha$. Because in general (for $\delta \approx 0.1$) $\Delta V^i_C/m^i \approx \mathcal {O}(10^{-4})$ the dependence of *M* and $\mu$ on $\alpha$ is expected to be rather small ($\Delta \mu /\mu \approx \mathcal {O}(10^{-4})$), whereas the change in the *Q*-value can be appreciable, $\Delta Q/Q \approx \mathcal {O}(10^{-1})$. Accordingly, the effect of a variation of $\mu$ with a variation of $\alpha$ on the value of $k_C=Z_v\,Z_c\,\mu c^2\,\alpha$ will be ignored.

We call the total of these kinematical variations the “indirect effect”.

In Ref. [1], we introduced an approximation to the dependence of the rate on $\alpha$ by evaluating the effect on the parameterized *S*-factor at an energy where the *S*-factor is supposed to be maximal. For charged particle induced reactions this energy is given by

54 $$\begin{aligned} \overline{E}=\left( \frac{k_B T}{2}\right) ^{\frac{2}{3}}\left( E_G^i\right) ^{\frac{1}{3}}. \end{aligned}$$

This then leads to a temperature dependent factor, that gives a fair approximation to parameterized results, both with and without including the indirect effects.

**Fig. 9:**
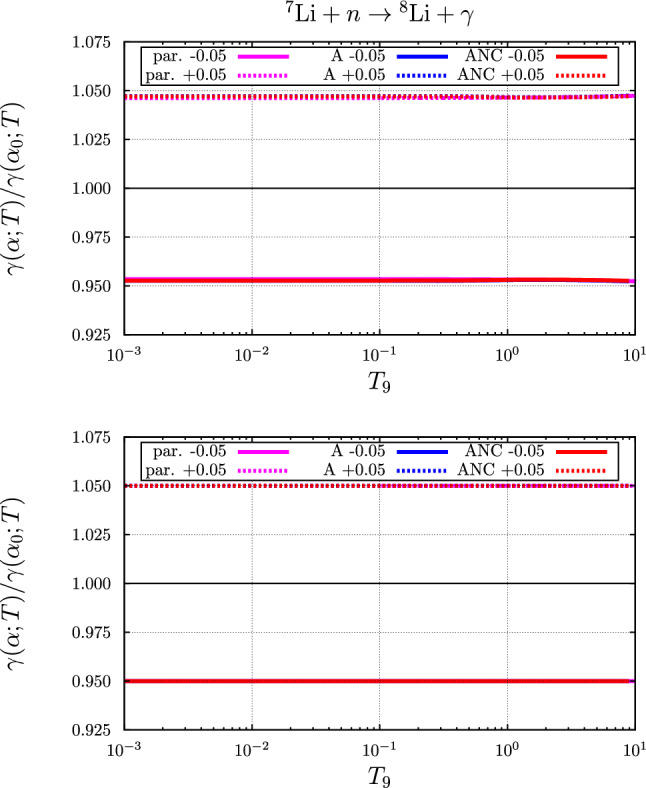
Variation of the temperature ($T_9 = T / 10^9~\text {K}$) dependent rate for the $n+ ^{7}{\text {Li}} \rightarrow  ^{8}{\text {Li}} + \gamma$ reaction with $\alpha =\alpha _0(1+\delta )$. The solid curves correspond to $\delta =-0.05$, the dotted lines to $\delta =+0.05$. Top panel: including both the direct and indirect effects; bottom panel: including the direct effect only

**Fig. 10:**
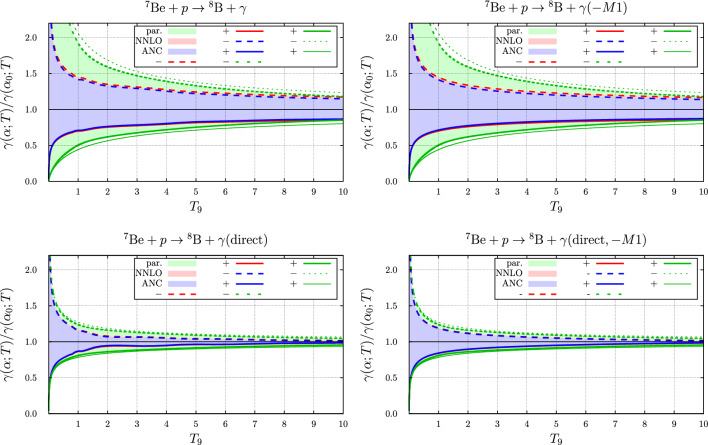
Variation of the rate normalized to the rate at the nominal $\alpha _0$ with varying $\alpha$, i.e. $\gamma (T_9;\alpha _0(1+\delta ))/\gamma (T_9;\alpha _0)$ for the $p+ ^{7}{\text {Be}} \rightarrow  ^{8}{\text {B}} + \gamma$ reaction. Here, $T_9 = T / 10^9~\text {K}$. Top left: total rate, including the *M*1 contribution. Top right: total rate, without the *M*1 contribution. Bottom left: direct variation only, including the *M*1 contribution. Bottom right: direct variation only, without the *M*1 contribution. The coloured areas bounded by the curves correspond to a variation $\delta \in [-0.05,+0.05]$. The green curves (areas) correspond to the variation of the rates with $\alpha$ according to the penetration factor and the trivial linear dependence of the cross section on $\alpha$ for dominant dipole radiation (direct effects). The blue and red curves (areas) represent the results for the parameter sets “ANC” and “NNLO”. The solid lines (marked ’+’) correspond to ${\delta }=0.05$, the dotted lines to ${\delta }=-0.05$. The thinner lines represent the results from the approximation to the fine-structure constant dependence of the rate based on the temperature-dependent factor evaluated at the energy $\overline{E}$ given in Eq. (54)

### The $n +  ^{7}{\text {Li}} \rightarrow  ^{8}{\text {Li}} + \gamma$ reaction

Since the neutron in the entrance channel is uncharged, there is no Coulomb interaction between the clusters and accordingly the direct effect of varying $\alpha$-dependence is completely determined by the fact that the cross section is strictly linear in $\alpha$. In addition there is the indirect effect stemming from the fine-structure constant dependence of the Coulomb contributions to the bindings energies of $^{7}{\text {Li}}$ and $^{8}{\text {Li}}$, that affects the *Q*-value of the reaction.

The variation of the rate with $\alpha$ is displayed in Fig. 9, where the relative variation $\gamma (\alpha ;T)/\gamma (\alpha _0;T)$ is plotted as a function of the temperature. Although the calculated rates, see Fig. 2, do differ slightly the relative changes of the rates are almost identical. The bottom panel in Fig. 9 indeed merely reflects that the cross section for this reaction trivially linearly depends on $\alpha$, i.e. if $\alpha$ varies by 5%, then also the direct effect rate varies by 5% and this effect is temperature-independent. Small deviations occur if also the variation of the binding energies of the Li-nuclides is taken into account, see the top panel of Fig. 9.

### The $p +  ^{7}{\text {Be}} \rightarrow  ^{8}{\text {B}} + \gamma$ reaction

The fine-structure constant dependence of the temperature dependent rate of the proton-induced radiative capture reaction is more interesting. In Fig. 10 the variation of the temperature-dependent rate with $\alpha$ of the calculated values with the two parameter sets is compared to the values obtained with the parameterization of the $\alpha$-dependence of the rates based on the parameterized cross sections as done in Ref. [1]. From this figure one infers that the relative variation in the Halo-EFT calculations is smaller by about $40\%$ than that found in Ref. [1]. Excluding the *M*1 contribution yields practically identical results. Furthermore, it is observed that considering also the indirect effect, i.e. also the effect on the binding energies and thus on the *Q*-value of the reaction, enhances this difference.

### The $^{3}{\text {H}} +  ^{4}{\text {He}} \rightarrow  ^{7}{\text {Li}} + \gamma$ reaction

**Fig. 11:**
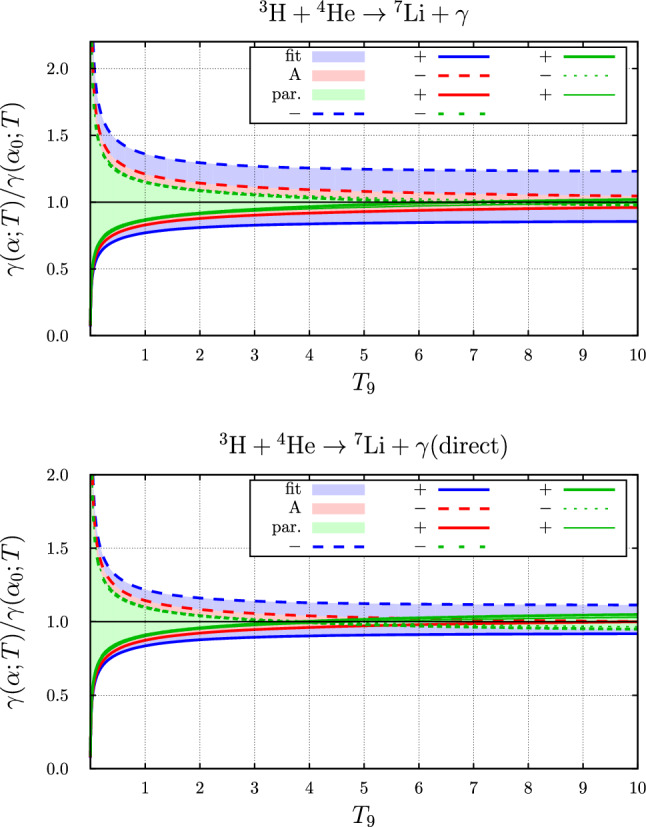
Fine-structure constant dependence of the temperature dependent rate of the $^{3}{\text {H}}+ ^{4}{\text {He}}\rightarrow  ^{7}{\text {Li}}+\gamma$ reaction. Shown is the rate relative to the rate with the nominal value: “+” is the rate at $\alpha =1.05\,\alpha _0$, “−” the value at $\alpha =0.95\,\alpha _0$. Here, $T_9 = T / 10^9~\text {K}$. The blue ranges were obtained with the parameter set “fit”, the red range with the parameter set “A” and the green range represents the variation of $\alpha$ as determined in Ref. [1]. The thinner lines represent the results from the approximation to the fine-structure constant dependence of the rate based on the temperature-dependent factor evaluated at the energy $\overline{E}$ given in Eq. (54)

The relative variation with $\alpha$ of the temperature-dependent rate for the two parameter sets “fit” and “A” are compared to that with the rate based on the parameterization of Ref. [1] in Fig. 11. Contrary to the previous reaction this variation is larger for the Halo-EFT results, in particular for the parameter set “fit” than that with the parameterized rate. Considering the direct effect only (bottom panel) leads to the same conclusion.

### The $^{3}{\text {He}} +  ^{4}{\text {He}} \rightarrow  ^{7}{\text {Be}} + \gamma$ reaction

**Fig. 12:**
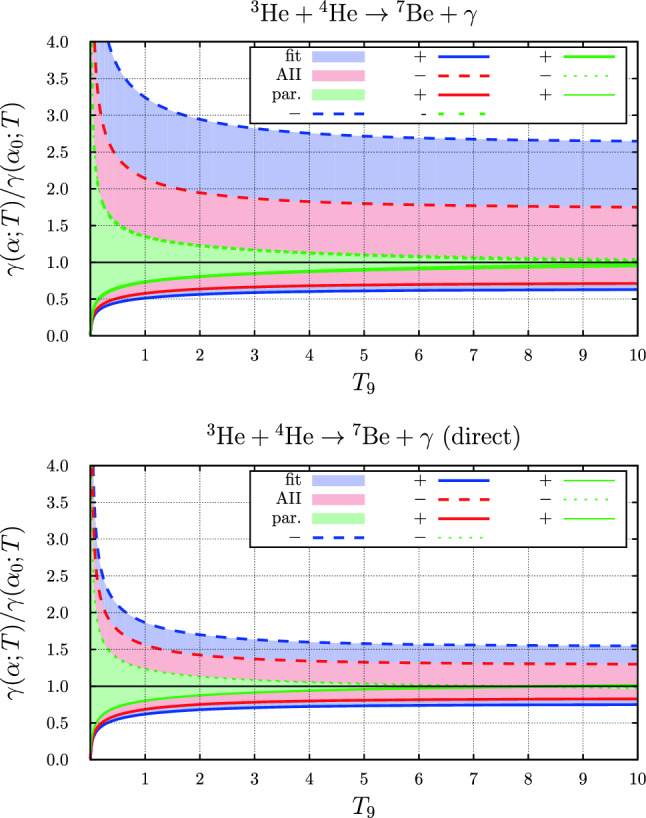
Fine-structure constant dependence of the temperature dependent rate of the $^{3}{\text {He}}+ ^{4}{\text {He}}\rightarrow  ^{7}{\text {Be}}+\gamma$ reaction. Here, $T_9 = T / 10^9~\text {K}$. Shown is the rate relative to the rate with the nominal value: “+” is the rate at $\alpha =1.05\,\alpha _0$, “−” the value at $\alpha =0.95\,\alpha _0$. The blue ranges were obtained with the parameter set “fit”, the red range with he parameter set “AII” and the green range represents the variation of $\alpha$ as determined in Ref. [1]. The thinner lines represent the results from the approximation to the fine-structure constant dependence of the rate based on the temperature-dependent factor evaluated at the energy $\overline{E}$ given in Eq. (54)

The relative variation of the rate with the value of the fine-structure constant $\alpha$ of the temperature-dependent rates for the two parameter sets “fit” and “AII” are compared to that with the rate based on the parameterization of Ref. [1] in Fig. 12. Here, the relative variation with the two Halo-EFT parameter sets is much larger than for the parameterization used previously, in particular if the fine-structure constant is smaller than the nominal value. We shall discuss the reason for this in the next section, Sect. 4.5.

### Discussion

**Fig. 13:**
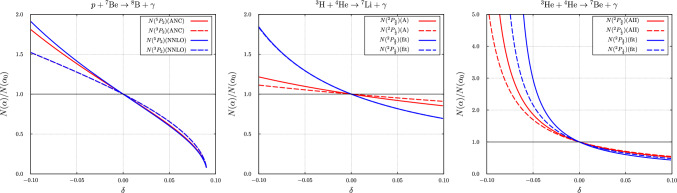
Variation of the relative normalization $$N(\alpha )/N(\alpha _0)$$ with $$\alpha = \alpha _0(1+\delta )$$ for the reactions $$p+ ^{7}{\text {Be}} \rightarrow  ^{8}{\text {B}} + \gamma $$ (left), $$ ^{3}{\text {H}}+ ^{4}{\text {He}} \rightarrow  ^{7}{\text {Li}} + \gamma $$ (middle), $$ ^{3}{\text {He}}+ ^{4}{\text {He}} \rightarrow  ^{7}{\text {Be}} + \gamma $$ (right). For the first reaction the results are shown for both the $$ ^{5}{P}_{2}$$ and the $$ ^{3}{P}_{2}$$ amplitudes contributing with equal weight to the ground state capture. For the other two reactions the normalisation of the $$ ^{2}{P}_{\frac{3}{2}}$$ (ground state) and the $$ ^{2}{P}_{\frac{1}{2}}$$ (excited state) are shown. The first reaction ceases to be exo-energetic for positive $$\delta \approx 0.1$$. For the last reaction the expression for the normalisation, see Eq. (13), exhibits a pole for negative $$\delta \approx -0.06$$

First of all we observe that although the resonant magnetic dipole contribution in the nucleon induced reaction accounts for a prominent feature in the cross sections (or astrophysical *S*-factors), this contribution is of minor importance in the variation of the rates with $$\alpha $$, see e.g. Fig. 10. Below we shall therefore focus on the effects from the dominant electric dipole contributions. With the exception of the neutron induced reaction the effects of the $$\alpha $$-variation differ from what was estimated on the basis of the parameterization of the cross-section used before in Ref. [1]. The dominant effect from the electric dipole contribution seems to be the $$\alpha $$ variation of the normalisation of Eq. (13). The variation with $$\alpha $$ of the relative normalisation $$N(\alpha )/N(\alpha _0)$$ for the three charged particle induced reactions is displayed in Fig. 13. For the proton induced reaction the results are shown for both the $$ ^{5}{P}_{2}$$ and the $$ ^{3}{P}_{2}$$ amplitudes contributing with equal weight to the ground state capture. For the other two reactions the normalisation of the $$ ^{2}{P}_{\frac{3}{2}}$$ (ground state) and the $$ ^{2}{P}_{\frac{1}{2}}$$ (excited state) are shown. This figure illustrates the main effects observed in the variation of the rates with $$\alpha $$:

1. Because of the absence of Coulomb interactions the variation with $\alpha$ of the cross sections and corresponding rates of the neutron induced reaction is trivially linear.
2. For the proton induced reaction the normalisation varies with $\alpha$ almost linearly by $\pm 40\%$ for $\delta \in [-0.05,0.05]$. Furthermore, the results for the two parameter sets considered here are almost identical. The variation of the rates is slightly larger for negative $\delta$, while considering the direct effect alone leads to a variation symmetric in $\delta$, in accordance with the variation of the normalisation. Also displayed in Fig. 10 is the variation of the parameterized rate with $\alpha$ on the basis of the approximation introduced in Ref. [1], by evaluating the effects at a fixed energy, see also Eq. (54). Indeed for the proton induced reaction this temperature dependence is larger than the result calculated in Halo-EFT in both cases.
3. In case of the $^{3}{\text {H}}$ and $^{3}{\text {He}}$ induced reactions the results with the approximation discussed above almost coincide with the results on the basis of the parameterization, as was already demonstrated in Ref. [1] and indeed in these cases is much smaller than what is to be expected on the basis of the normalisation. Anyhow, the $\alpha$ dependence of the normalisation is rather asymmetric in $\delta$ as shown in Fig. 13. For the $^{3}{\text {He}}$ induced reaction this is even more prominent since the denominator in the expression for the norm, see Eq. (13), vanishes for $\delta < -0.06$, corresponding to a pole in the normalisation and thus leading to a very asymmetric $\delta$ dependence in this case.
4. Accordingly, within Halo-EFT the study of the $\alpha$ dependence of the rates is limited to a rather moderate relative variation of $\alpha$ of $5\%$ only.

**Table 4:** Nominal abundances as number ratios $Y_n/Y_H$ (for $^4\text {He}$ the mass ratio $Y_p$) calculated with the modified versions of the codes as in Ref. [1], but with the nominal results (i.e. $\alpha =\alpha _0$) for the four reactions considered in this work. The value of the baryon-to-photon ratio and the nominal value of the neutron lifetime are $\eta = 6.14 \cdot 10^{-10}$ and $\tau _n = 879.4\,\text {s}$, respectively. For comparison also the values previously obtained in Ref. [1] are listed

code	$^{2}{\text {H}}$	$^{3}{\text {H}}+ {\text {He}}$	$Y_{p}$	$^{6}{\text {Li}}$	$^{7}{\text {Li}}+ {\text {Be}}$
	$\times 10^5$	$\times 10^5$		$\times 10^{14}$	$\times 10^{10}$
NUC123	2.500	1.139	0.246	1.808	5.540
[1]	2.501	1.139	0.246	1.809	5.172
PArthENoPE	2.569	1.147	0.247	1.819	5.376
[1]	2.569	1.147	0.247	1.820	5.017
AlterBBN	2.585	1.153	0.248	1.903	5.350
[1]	2.585	1.153	0.248	1.904	4.993
PRIMAT	2.562	1.150	0.247	1.861	5.394
[1]	2.563	1.149	0.247	1.862	5.033
PRyMordial	2.581	1.148	0.247	1.891	5.448
PDG [13]	2.547		0.245		1.6
$\qquad \pm$	0.025		0.003		0.3

## Abundances

In order to assess the relevance of the electromagnetic fine-structure constant dependence of the reaction rates for the resulting abundances of the light elements in BBN, we evaluated these abundances with five different publicly available codes, viz. NUC123 [14], PArthENoPE [19], AlterBBN [16], PRIMAT [20] and PRyMordial [21, 22]. We use the rates as in our previous work, see [1], substituting the $\alpha$ dependence of the rates for the four reactions considered here as discussed above. More specifically, for calculating the $\alpha$ dependence of the abundances, we used the parameter sets “NNLO”, “fit” and “fit” for the $p +  ^{7}{\text {Be}} \rightarrow  ^{8}{\text {B}} + \gamma$, $^{3}{\text {H}} +  ^{4}{\text {He}} \rightarrow  ^{7}{\text {Li}} + \gamma$ and $^{3}{\text {He}} +  ^{4}{\text {He}} \rightarrow  ^{7}{\text {Be}} + \gamma$ reactions, respectively, the $\alpha$-dependence of neutron induced radiative capture reaction $n +  ^{7}{\text {Li}} \rightarrow  ^{8}{\text {Li}} + \gamma$ being practically linear anyway. As was demonstrated in the Sect. 4 these parameter sets showed the largest variation of the rates with $\alpha$.

**Fig. 14:**
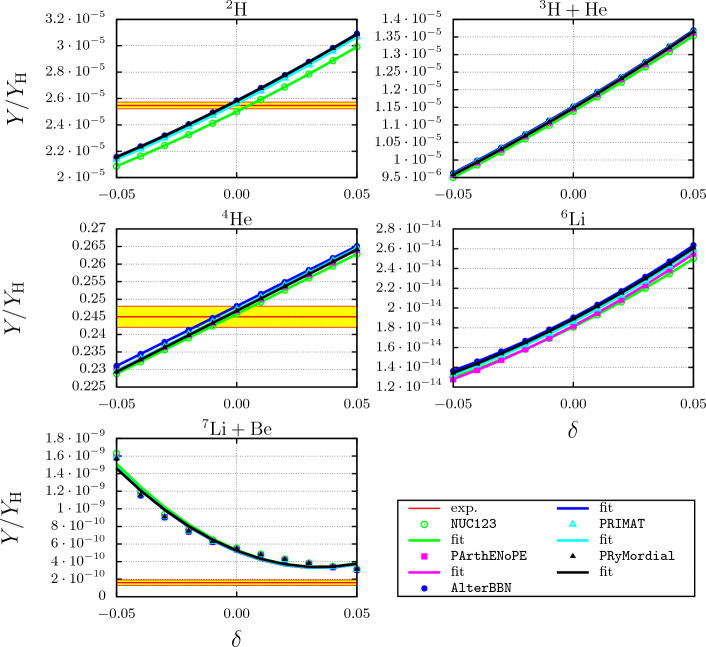
Variation of the abundance ratios $Y_n/Y_H$ with a variation of $\alpha = \alpha _0\,(1+\delta )$ for $\delta \epsilon \in [-0.05,0.05]$ obtained with the codes: NUC123 [14], AlterBBN [16], PArthENoPE [19], PRIMAT [20] and PRyMordial [21, 22]. Here, we use $\eta =6.14 \cdot 10^{-10}$ and $\tau _n = 879.4\,\text {s}$. Also shown are the solid curves obtained by the fits according to Eq. (55) with the parameters listed in Table 5. The experimental values cited in PDG [13] (thick red lines) are indicated by yellow-highlighted regions (color online) representing the $1\sigma$ limits by red lines

In Table 4 we list the nominal abundances, i.e. for $\alpha =\alpha _0$, see Eq. (40). The results show that with the exception of the values for the $^{7}{\text {Li}}+  {\text {Be}}$ abundance, which are larger by about $10\%$, and thus slightly deteriorate the so-called “Li-problem”, the treatment of the four reactions in Halo-EFT as considered here leads to results practically identical to those obtained previously in Ref. [1].

The fine-structure constant dependence of the primordial abundances is depicted in Fig. 14.

Again, the results for the $d-$, $^{3}{\text {H}}+  {\text {He}}-$, $^{4}{\text {He}}-$ and $^{6}{\text {Li}}-$ abundances are very similar to those obtained previously, see Fig. 5 in Ref. [1]. Moreover, the five BBN-codes considered here produce consistent results, in spite of the fact that these codes differ in details, such as the number of reactions in the BBN network or the manner in which the rate equations are solved numerically. This then also applies to the values for the resulting response matrix elements. The (linear) response matrix elements $\partial \log {(Y_n/Y_H)}/\partial \log {\alpha } = c_1$ and the coefficients of the quadratic term ($c_2$) in a quadratic least-squares fit of the form

55 $$\begin{aligned} P_k(\delta ) = c_0(1 + c_1 \delta + c_2 \delta ^2), \end{aligned}$$

are given and compared to the results obtained previously in Table 5.

We do find a very different result for the $\alpha$-dependence of the $^{7}{\text {Li}}+  {\text {Be}}$ abundance: In particular the linear response coefficient is approximately five times larger than the value obtained previously and moreover the response is far from linear, the quadratic coefficient being approximately 40 times larger than the value previously obtained in [1], as can also be seen from a comparison of Fig. 14 with Fig. 5 of Ref. [1].

If instead of the parameter sets “NNLO”, “fit” and “fit” for the reactions $p +  ^{7}{\text {Be}} \rightarrow  ^{8}{\text {B}} + \gamma$, $^{3}{\text {H}} +  ^{4}{\text {He}} \rightarrow  ^{7}{\text {Li}} + \gamma$ and $^{3}{\text {He}} +  ^{4}{\text {He}} \rightarrow  ^{7}{\text {Be}} + \gamma$, respectively, we use the parameter sets “ANC”, “A” and “AII” of Tables 2 and 3 for these three reactions, respectively, we find similar results, except for the $^{7}{\text {Li}}+  {\text {Be}}$ response coefficients: In accordance with the fact that, as was shown in Sect. 4, the change of the rates with $\alpha$ was found to be smaller for these parameters, the linear response coefficient $c_1$ is about half as large and the quadratic coefficient is smaller by a factor 2.5, still corresponding to an appreciable curvature.

**Table 5:** BBN response matrix $$c_1 = \partial \log {(Y_n/Y_H)}/\partial \log {\alpha }$$ and the coefficients $$c_2$$ of the quadratic term in Eq. (55) at $$\eta =6.14 \cdot 10^{-10}$$ and $$\tau _n = 879.4\,\text {s}$$. $$Y_n/Y_H$$ are the number ratios of the abundances relative to hydrogen; $$Y_p$$ is conventionally the $$ ^4$$He/H mass ratio. The results obtained with the five BBN codes NUC123 [14], PArthENoPE [19], AlterBBN [16], PRIMAT [20] and PRyMordial [21, 22] are compared to the results previously obtained in Ref. [1]. The odd rows show the results with the rates for the reactions evaluated in the present contribution for the reactions $$p +  ^{7}{\text {Be}} \rightarrow  ^{8}{\text {B}} + \gamma $$, $$ ^{3}{\text {H}} +  ^{4}{\text {He}} \rightarrow  ^{7}{\text {Li}} + \gamma $$ and $$ ^{3}{\text {He}} +  ^{4}{\text {He}} \rightarrow  ^{7}{\text {Be}} + \gamma $$ where the parameter sets “NNLO”, “fit” and “fit” were used, respectively. Otherwise the rates are identical to those in  [1]

Code	$^{2}{\text {H}}$		$^{3}{\text {H}}+ ^{3}{\text {He}}$		$Y_{p}$		$^{6}{\text {Li}}$		$^{7}{\text {Li}}+ ^{7}{\text {Be}}$	
	$c_1$	$c_2$	$c_1$	$c_2$	$c_1$	$c_2$	$c_1$	$c_2$	$c_1$	$c_2$
NUC123	3.620	6.198	3.539	4.661	1.386	0.035	6.642	20.074	$-$ 21.296	312.074
[1]	3.655	6.228	3.540	4.625	1.387	0.016	6.830	20.412	$-$ 4.325	7.480
PArthENoPE	3.606	6.173	3.534	4.619	1.390	0.056	6.968	21.116	$-$ 21.284	312.328
[1]	3.635	6.182	3.533	4.577	1.389	0.065	7.159	21.482	$-$ 4.308	7.715
AlterBBN	3.610	6.135	3.526	4.591	1.375	0.048	6.651	20.167	$-$ 21.312	312.939
[1]	3.644	6.188	3.526	4.568	1.373	0.049	6.857	20.499	$-$ 4.322	7.865
PRIMAT	3.627	6.253	3.535	4.631	1.415	0.072	6.754	20.593	$-$ 21.273	311.595
[1]	3.658	6.264	3.534	4.595	1.408	0.081	6.953	20.828	$-$ 4.302	7.563
PRyMordial	3.609	5.975	3.544	4.756	1.411	0.081	6.698	18.453	$-$ 20.694	300.083

## Summary

In this work we have studied the fine-structure constant dependence of some BBN-relevant radiative capture reactions within the framework of Halo-EFT. We concentrated on the main effects, refraining from implementing a coupled channel approach as would be dictated by strict EFT power counting. Nevertheless we studied for each reaction two parameter sets in order to obtain an indication of the systematic errors. We found that the effects do deviate from what has been found previously on the basis of parameterized cross section data and a simple parameterization of the $\alpha$ dependence motivated by a simple penetration factor. While for a neutron induced radiative capture reaction the results are almost strictly linear, as is to be expected since the radiative capture reaction amplitude is linear in the electromagnetic coupling and thus the cross section is linear in $\alpha$, for charged particle reactions the direct effect can both be smaller, as is the case for the $^{7}{\text {Be}}(p,\gamma ) ^{8}{\text {B}}$ reaction, or larger, as is the case for the $^{4}{\text {He}}( ^{3}{\text {H}},\gamma ) ^{7}{\text {Li}}$ and the $^{4}{\text {He}}( ^{3}{\text {He}},\gamma ) ^{7}{\text {Be}}$ radiative captures, than what is to be expected on the basis of the parameterized treatment.

In spite of these substantial deviations from the $\alpha$-dependence of the parameterized rates obtained for these reactions previously, the impact on the resulting abundances and on their $\alpha$-dependence of the light elements $^{2}{\text {H}}$, $^{3}{\text {H}}+  {\text {He}}$, $^{4}{\text {He}}$, $^{6}{\text {Li}}$ with the rates calculated within the framework of Halo-EFT is very minor only. In contrast, for the $^{7}{\text {Li}}+  {\text {Be}}$-abundance we do find that the $\alpha$-dependence differs appreciably from that of the previous parameterized results, this $\alpha$-dependence being much more pronounced and clearly non-linear with the Halo-EFT rates. Also the nominal abundance (i.e. calculated with the current value of the fine-structure constant $\alpha _0$) of $^{7}{\text {Li}}+  {\text {Be}}$ is larger by almost 10 %, whereas the other abundances remain practically unchanged.

For reactions involving charged particles, the Halo-EFT calculation accounts for the charged particle repulsion by inclusion of the full Coulomb propagator in all reaction steps. As the present study shows, these Coulomb effects cannot always be approximated by a universal penetration factor. It was also found that in some cases the study of the fine-structure dependence of cross sections and the corresponding rates within the framework of Halo-EFT can be limited by singularities appearing in the normalisation, that enters as a factor in the resulting cross sections. This was found to be relevant for the $^{3}{\text {He}} +  ^{4}{\text {He}} \rightarrow  ^{7}{\text {Be}} + \gamma$ reaction, limiting the study to relative variations of $\alpha$ smaller than $6\%$. Furthermore, it should be stressed that the Halo-EFT framework is of course restricted to those reactions where the di-nuclear structure assumption underlying this is indeed applicable. Therefore a definite assessment of the fine-structure dependence of rates relevant for primordial nucleosynthesis should ultimately be performed within a framework that allows for a genuine ab initio treatment of nuclear reaction dynamics. Indeed recent progress within the framework of nuclear lattice effective field theory (NLEFT), see e.g. Ref. [64], shows that NLEFT seems to be a promising candidate for such a treatment.

## Data Availability

Data will be made available on reasonable request. [Authors’ comment: The datasets generated during and/or analysed during the current study are available from the corresponding author on reasonable request.]

## Appendix A: *C*-integral

To calculate

A1 $$\begin{aligned} C(p)= &   \lim _{\delta \downarrow 0}\, \frac{\mu ^2}{6\,\pi ^2\,(p^2+\gamma ^2)} \nonumber \\  &   \times \int _{0}^{1}\!\!\!\text {d}{x} \int _{0}^{1}\!\!\!\text {d}{y} \frac{1}{\sqrt{x\,(1-x)}} \frac{1}{\sqrt{1-y}} \nonumber \\  &   \times \Biggl ( x\,p^2\, \log {\left[ \frac{\pi }{4\,k_C^2}\left( -y\,p^2+(1-y)\frac{\gamma ^2}{x}-\text {i}\,\delta \right) \right] } \nonumber \\  &   + p^2\, \log {\left[ \frac{\pi }{4\,k_C^2}\left( -y\,p^2-(1-y)\frac{p^2}{x}-\text {i}\,\delta \right) \right] } \nonumber \\  &   + x\,\gamma ^2\, \log {\left[ \frac{\pi }{4\,k_C^2}\left( y\,\gamma ^2+(1-y)\frac{\gamma ^2}{x}-\text {i}\,\delta \right) \right] } \nonumber \\  &   + \gamma ^2\, \log {\left[ \frac{\pi }{4\,k_C^2}\left( y\,\gamma ^2-(1-y)\frac{p^2}{x}-\text {i}\,\delta \right) \right] } \Biggr ).\nonumber \\ \end{aligned}$$

Because the integral over *y* can be performed analytically this reduces to a single integral and, with the substitution $x=\sin ^2{\vartheta }$, one obtains with $\kappa = \gamma /p$ and $\eta _p = k_C/p$ :

A2 $$\begin{aligned} \frac{C(p)}{\mu ^2} = C_0 + C_1(\kappa ), \end{aligned}$$

where

A3 $$\begin{aligned} C_0 = \frac{1}{2\pi }\,\log \left[ \frac{\pi }{4\,\eta _p^2}\right] \end{aligned}$$

and

$$\begin{aligned}  &   \!\!\! C_1(\kappa ) =\frac{1}{3\pi ^2\left( 1+\kappa ^2\right) }\\  &   \quad \int _{0}^{\frac{\pi }{2}}\!\!\!\text {d}{\vartheta } \Bigl \lbrace \sin ^2{\vartheta }\,c_1(\kappa ;\sin ^2{\vartheta })+ c_2(\kappa ;\sin ^2{\vartheta })\\  &   \qquad +\sin ^2{\vartheta }\,\kappa ^2\,c_3(\kappa ;\sin ^2{\vartheta })+\kappa ^2\,c_4(\kappa ;\sin ^2{\vartheta })\Bigr \rbrace , \end{aligned}$$

to be evaluated numerically with the integrands

A4 $$\begin{aligned} c_1(\kappa ;x)= &   \int _{0}^{1}\!\!\!\text {d}{y}\,\,\frac{1}{\sqrt{1-y}}\,\log {\left[ -y + (1-y)\,\frac{\kappa ^2}{x}-\text {i}\,\delta \right] } \nonumber \\= &   2\,\log {\left[ \frac{\kappa ^2}{x}\right] } - 4 \nonumber \\  &   + \frac{2}{\sqrt{\frac{\kappa ^2}{x}+1}} \left\{ \log {\left[ \frac{\sqrt{\frac{\kappa ^2}{x}+1}+1}{\sqrt{\frac{\kappa ^2}{x}+1}-1} \right] } -\text {i}\,\pi \right\} , \nonumber \\ \end{aligned}$$

A5 $$\begin{aligned} c_2(\kappa ;x)= &   \int _{0}^{1}\!\!\!\text {d}{y}\,\,\frac{1}{\sqrt{1-y}}\,\log {\left[ -y - (1-y)\,\frac{1}{x}-\text {i}\,\delta \right] } \nonumber \\= &   -2\,\log {\left[ x\right] } - 2\,\text {i}\,\pi - 4 \nonumber \\  &   + 4\,\sqrt{\frac{x}{1-x}}\, \arctan {\left( \sqrt{\frac{1-x}{x}}\right) }, \end{aligned}$$

A6 $$\begin{aligned} c_3(\kappa ;x)= &   \int _{0}^{1}\!\!\!\text {d}{y}\,\,\frac{1}{\sqrt{1-y}}\,\log {\left[ \kappa ^2\,y + (1-y)\,\frac{\kappa ^2}{x}-\text {i}\,\delta \right] } \nonumber \\= &   2\,\log {\left[ \frac{\kappa ^2}{x}\right] } - 4 \nonumber \\  &   + 4\,\sqrt{\frac{x}{1-x}}\, \arctan {\left( \sqrt{\frac{1-x}{x}}\right) }, \end{aligned}$$

A7 $$\begin{aligned} c_4(\kappa ;x)= &   \int _{0}^{1}\!\!\!\text {d}{y}\,\,\frac{1}{\sqrt{1-y}}\,\log {\left[ \kappa ^2\,y - (1-y)\,\frac{1}{x}-\text {i}\,\delta \right] } \nonumber \\= &   -2\,\log {\left[ x\right] } - 2\,\text {i}\,\pi - 4 \nonumber \\  &   + \frac{2\,\kappa }{\sqrt{\frac{1}{x}+\kappa ^2}} \left\{ \log {\left[ \frac{\sqrt{\frac{1}{x}+\kappa ^2}+\kappa }{\sqrt{\frac{1}{x}+\kappa ^2}-\kappa } \right] } +\text {i}\,\pi \right\} . \nonumber \\ \end{aligned}$$

## Appendix B: $D'$-integral

To calculate

B1 $$\begin{aligned}  &   D'(k_C;p,\gamma ) = \lim _{\delta \downarrow 0}\, \frac{\mu ^2}{6\,\pi ^2\,(p^2+\gamma ^2)} \nonumber \\  &   \quad \times \int _{0}^{1}\!\!\!\text {d}{x} \int _{0}^{1}\!\!\!\text {d}{y} \sqrt{\frac{x}{1-x}} \frac{1}{\sqrt{1-y}} \nonumber \\  &   \quad \times \Biggl ( p^2\, \log {\left[ \frac{1}{4\,k_C^2}\left( -y\,p^2+(1-y)\frac{\gamma ^2}{x}-\text {i}\,\delta \right) \right] } \nonumber \\  &   \qquad + p^2\, \log {\left[ \frac{1}{4\,k_C^2}\left( -y\,p^2-(1-y)\frac{p^2}{x}-\text {i}\,\delta \right) \right] } \nonumber \\  &   \qquad + \gamma ^2\, \log {\left[ \frac{1}{4\,k_C^2}\left( y\,\gamma ^2+(1-y)\frac{\gamma ^2}{x}-\text {i}\,\delta \right) \right] } \nonumber \\  &   \qquad + \gamma ^2\, \log {\left[ \frac{1}{4\,k_C^2}\left( y\,\gamma ^2-(1-y)\frac{p^2}{x}-\text {i}\,\delta \right) \right] } \Biggr ).\nonumber \\ \end{aligned}$$

Thus, with $\eta _p={k_C}/{p}$ and $\kappa ={\gamma }/{p}={\eta _p}/{\eta _\gamma }$ :

$$\begin{aligned} \frac{D'(\eta _p,\kappa )}{\mu ^2}= &   \lim _{\delta \downarrow 0}\,\frac{1}{6\,\pi ^2\,(1+\kappa ^2)}\nonumber \\  &   \times \int _{0}^{1}\!\!\!\text {d}{x}\int _{0}^{1}\!\!\!\text {d}{y}\sqrt{\frac{x}{1-x}}\frac{1}{\sqrt{1-y}}\nonumber \\  &   \times \Biggl ( \log {\left[ \frac{1}{4\,\eta _p^2}\left( -y + (1-y)\frac{\kappa ^2}{x}-\text {i}\,\delta \right) \right] } \nonumber \\  &   + \log {\left[ \frac{1}{4\,\eta _p^2}\left( -y - (1-y)\frac{1}{x}-\text {i}\,\delta \right) \right] } \nonumber \\  &   + \kappa ^2\, \log {\left[ \frac{1}{4\,\eta _p^2}\left( y\,\kappa ^2 + (1-y)\frac{\kappa ^2}{x}-\text {i}\,\delta \right) \right] } \nonumber \\  &   + \kappa ^2\, \log {\left[ \frac{1}{4\,\eta _p^2}\left( y\,\kappa ^2 - (1-y)\frac{1}{x}-\text {i}\,\delta \right) \right] } \Biggr ), \end{aligned}$$

i.e

B2 $$\begin{aligned} \frac{D'(\eta _p,\kappa )}{\mu ^2}= &   -\frac{1}{3\,\pi }\,\log {\left( 4\,\eta _p^2\right) }\nonumber \\  &   +\frac{1}{3\,\pi ^2\,(1+\kappa ^2)}\int _{0}^{\frac{\pi }{2}}\!\!\!\text {d}{\vartheta }\sin ^2\vartheta \nonumber \\  &   \times \Bigl \lbrace c_1(\kappa ,\sin ^2\vartheta ) + c_2(\kappa ,\sin ^2\vartheta ) \nonumber \\  &   + \kappa ^2\,c_3(\kappa ,\sin ^2\vartheta ) + \kappa ^2\,c_4(\kappa ,\sin ^2\vartheta ) \Bigr \rbrace . \end{aligned}$$

in terms of the integrands of Eqs. A4–A7 of Appendix A.

## Footnotes

1 As pointed out in Ref. [8] the divergent pieces of *B* cancel the divergent pieces in $J_0$, this was accounted for in arriving at Eq. (19).

2 The partial derivative involved might be difficult to find analytically. Although in principle not particularly stable, one could use numerical approximations to the (partial) derivative of a function $\mathcal {B}(\kappa ,\eta _\gamma ;\rho )$, such as (with $\mathcal {B}_j = \mathcal {B}(\kappa ,j\,h;\rho )$):

$$\begin{aligned}  &   \left. (\partial _{\eta _\gamma } \mathcal {B}(\kappa ,\eta _\gamma ,\rho ) \right| _{\eta _\gamma =0} \\  &   \quad =\frac{8\,(\mathcal {B}_{+1}-\mathcal {B}_{-1})-(\mathcal {B}_{+2}-\mathcal {B}_{-2}))}{12\,h}+\mathcal {O}(h^4)\\  &   \quad =\frac{45\,(\mathcal {B}_{+1}-\mathcal {B}_{-1})-9\,(\mathcal {B}_{+2}-\mathcal {B}_{-2})+(\mathcal {B}_{+3} - B_{-3}) }{60\,h}+\mathcal {O}(h^6) \end{aligned}$$

for some small finite *h*.

3 Note that the current due to the velocity of fragment *i* is given by the operator $(Z_i\,e)/(m_i)\,\widehat{\textbf{p}}_i$. Accordingly, the associated orbital magnetic moment, expressed in units of $\mu _N = (e\,\hbar c)/(2\,m_p c^2)$, reads $\mu _i = Z_i\,(m_p/m_i)\,\mu _n\,\widehat{\textbf{L}}_i$ with $L_i$ the angular momentum of fragment *i*.

## Code Availability Statement

Code/software will be made available on reasonable request. [Authors’ comment: The code/software generated during and/or analysed during the current study is available from the corresponding author on reasonable request.]
